# Comparison of corneal endothelial cell changes after phacoemulsification between type 2 diabetic and nondiabetic patients

**DOI:** 10.1097/MD.0000000000027141

**Published:** 2021-09-03

**Authors:** Jin-Ho Joo, Tae Gi Kim

**Affiliations:** Department of Ophthalmology, Kyung Hee University Hospital at Gangdong, Kyung Hee University, Seoul, Republic of Korea.

**Keywords:** cataract, central corneal thickness, corneal endothelial cell, diabetes mellitus, phacoemulsification

## Abstract

The aim of this study is to compare the endothelial cell density (ECD) and morphology between diabetic mellitus (DM) and nondiabetic patients at 1 year after phacoemulsification in operated eyes and nonoperated eyes.

Evaluation was performed in 28 patients (56 eyes) with type 2 diabetes and 37 patients (74 eyes) without diabetes who underwent 1-year interval cataract surgery. Using a noncontact specular microscope and Scheimpflug rotating camera, corneal parameters were analyzed before and 1 year after surgery. Subgroups analysis was performed based on a disease duration 10 years and HbA1c concentration 7% and Pearson correlation analysis was performed.

The mean change in ECD at 1 year after surgery was 13.28% in the DM group and 11.40% in the control group. In the fellow nonoperated eyes, the mean change was 4.47% and 3.63% in the DM and control groups, respectively. There was no significant difference in postoperative ECD, coefficient of variance, hexagonality, and central corneal thickness between 2 groups. In the subgroup analysis, the long disease duration DM group (≥10 years) had a significantly greater ECD loss than the control and short disease duration DM groups (<10 years). Blood urea nitrogen (BUN) showed a significant correlation with postoperative ECD change (*r* = −0.474, *P* = .011).

The diabetic group with a longer disease duration showed significantly greater ECD decrease compared to the nondiabetic group and BUN correlated with ECD changes after phacoemulsification. Postoperative ECD loss may be high if the disease duration is long or if the BUN level is high.

## 1 Introduction

Cataract is the leading cause of blindness worldwide.^[1]^ Phacoemulsification is the most common surgical procedure for the treatment of cataracts and can improve visual acuity with few complications.^[2,3]^ Despite the potential advantages of phacoemulsification, corneal endothelial cell loss can occur due to injury from ultrasound energy during surgery. Therefore, corneal endothelial cell damage remains a major concern after phacoemulsification. Since the corneal endothelial cell density (ECD) is known to decrease with age, endothelial cell damage can be significant in older people. In addition to age, systemic diseases such as diabetes can also affect the corneal endothelium.

The mechanism by which diabetes causes corneal endothelial cell damage has not been clearly identified, but several hypotheses have been proposed. In diabetes, oxidative stress can occur due to the accumulation of advanced glycation end products in corneal endothelial cells.^[4]^ Diabetes also decreases Na^+^K^+^-ATPase activity in endothelial cells that play a role in maintaining the endothelial structure.^[5]^ These mechanisms can reduce corneal endothelial cell function.

Several studies have shown that diabetic patients may be more susceptible to corneal complications before and after intraocular surgery.^[6–26]^ According to the reported results, after cataract surgery, the loss of ECD in diabetic patients was 5.95% to 29.07%, compared to 0.88% to 18.18% in healthy individuals.^[6–17]^ Previous studies have shown varying results on whether diabetic patients have an increased risk of ECD loss after phacoemulsification. In addition, most of the studies evaluated short-term changes within 3 months, and there are few papers analyzing the effect of diabetic duration and results from serum laboratory tests such as HbA1c and blood urea nitrogen (BUN) on ECD loss.^[9,17]^ Since loss of corneal endothelial cells can occur naturally over time, it is important to compare both the operated and the nonoperated eyes.

The purpose of this study was to compare the 1-year change of ECD, coefficient of variance (CV), hexagonality, central corneal thickness (CCT) in operated eyes and their nonoperated eyes in diabetic and control patients who underwent 1-year interval cataract surgery. Subgroup analysis was performed according to the diabetes duration and HbA1c concentration. Moreover, we aimed to determine the association of ECD loss with laboratory data, including ocular and systemic parameters such as disease duration, HbA1c, BUN, and serum creatinine.

## 2 Methods

### 2.1 Study design and patients

This retrospective study included 130 eyes of 65 patients (56 diabetic and 74 nondiabetic eyes) who underwent 1-year interval phacoemulsification at the Kyung Hee University Hospital at Gangdong, Seoul, Korea, between January 2016 and December 2019 by 2 experienced surgeons using a standard technique with the same phacoemulsification machine. The inclusion criterion was grade II or III cataract according to the Lens Opacity Classification System III. Patients with a history of ocular surgery, dry eye disease, uveitis or ocular inflammation, corneal opacities, glaucoma, and ocular trauma were excluded from the study. Patients who underwent cataract surgery were divided into the diabetic mellitus (DM) and control groups, and the nonoperated were also analyzed by being dividing into the DM and control groups. The diagnosis of diabetes was based on the medical records of those under medical treatment for glucose control. We performed a subgroup analysis for diabetic patients with diabetes duration greater and less than 10 years and HbA1c greater and less than 7%. This study was approved by the Institutional Review Board of Kyung Hee University Hospital at Gangdong (approval number: 2020-10-017). Informed consent was waived because of the retrospective nature of the study and the analysis used anonymous clinical data.

### 2.2 Patient examinations

Preoperatively, all patients underwent complete ophthalmic evaluation, including slit-lamp examination, noncontact tonometer, and posterior segment examination with pupil dilation. ECD, percentage of hexagonal cells, and CV in cell size were evaluated preoperatively and 1 year postoperatively using a Topcon SP 3000P Specular Microscope (Topcon Corporation, Tokyo, Japan) in all patients. CCT was measured using a Scheimpflug rotating camera (Pentacam, Oculus, Wetzlar, Germany). Axial length and anterior chamber depth were measured using A-scan (Aviso, Quantel Medical, Clermont-Ferrand, France). Serum samples of patients taken before surgery were analyzed to evaluate HbA1c, BUN, and creatinine levels.

### 2.3 Surgical technique

All procedures were performed under topical anesthesia. Phacoemulsification and implantation of an intraocular lens were performed by 2 experienced surgeons via a 2.80 mm sutureless clear corneal incision with Sovereign Compact Cataract Extraction System (Abbott Medical Optics Inc., IL). The intraocular lens was placed within the capsular bag in all cases. Postoperatively, all patients received the same treatment regimen consisting of a combination of an antibiotic and a steroid.

### 2.4 Statistical analysis

Statistical analysis was performed using the independent samples *t* test in SPSS version 18.0 software (SPSS Inc., Chicago, IL). Comparisons of pre- and postoperative ECD, CV, hexagonality, and CCT between the 2 groups were calculated using an unpaired *t* test. Pearson correlation was used to analyze the correlation of ECD changes with the clinical characteristics of diabetic patients, including disease duration, HbA1c levels, serum glucose, serum urea nitrogen, and serum creatinine. A *P*-value of less than .05 was considered statistically significant.

## 3 Results

A total of 130 eyes of 65 patients (26 men, 39 women) were included in the study: 28 patients in the DM group and 37 patients in the control group. Preoperative demographics are shown in Table 1. There were no statistically significant differences between the 2 groups in terms of age, mean preoperated ECD, CV, hexagonality, and CCT (*P* > .05). HbA1c and blood glucose levels were significantly higher in the DM group than in the control group (*P* = .000). However, preoperative serum BUN and creatinine did not show statistically significant differences between the 2 groups.

**Table 1:** Baseline patient demographics.

	Operated eye		Fellow nonoperated eye			
	DM	Control	DM	Control	^∗^ *P*	^∗∗^ *P*
Sample size (eyes) (M:F)	28 (14: 14)	37 (12: 25)	28 (14: 14)	37 (12: 25)		
Age (yr)	63.18 ± 2.46	68.68 ± 1.37	63.18 ± 2.46	68.68 ± 1.37	.58	.58
Axial length (mm)	23.44 ± 0.91	23.48 ± 1.17	23.48 ± 0.89	23.62 ± 1.15	.873	.601
ACD (mm)	3.26 ± 0.57	3.06 ± 0.54	3.28 ± 0.56	3.02 ± 0.50	.151	.071
Preoperative ECD (cells/mm^2^)	2714.51 ± 402.711	2703.53 ± 265.97	2733.13 ± 304.51	2722.94 ± 259.24	.901	.887
Coefficient of variation	36.24 ± 6.64	34.31 ± 6.78	36.23 ± 6.97	34.93 ± 7.26	.256	.468
Hexagonal cell ratio (%)	53.75 ± 7.91	56.32 ± 13.48	53.14 ± 9.69	55.68 ± 13.06	.340	.373
Central corneal thickness (μm)	551.61 ± 44.26	546.54 ± 28.91	554.68 ± 42.38	548.35 ± 23.19	.601	.480
Nucleosclerosis (LOCS III)	2.77 ± 0.47	2.73 ± 0.64	–	–	.748	–
HbA1c	7.35 ± 1.61	5.62 ± 0.87	7.35 ± 1.61	5.62 ± 0.87	.000	.000
Blood glucose	156.36 ± 60.00	106.95 ± 16.82	156.36 ± 60.00	106.95 ± 16.82	.000	.000
Blood urea nitrogen	18.11 ± 7.37	15.99 ± 3.54	18.11 ± 7.37	15.99 ± 3.54	.155	.155
Creatinine	1.09 ± 0.98	0.77 ± 0.21	1.09 ± 0.98	0.77 ± 0.21	.099	.099

ACD = anterior chamber depth, DM = diabetic mellitus, ECD = endothelial cell density, LOCS III = Lens Opacities Classification System, version III.

∗ *P* value by independent *t* test between operated DM and operated control group.

∗∗ *P* value by independent *t* test between nonoperated DM and nonoperated control group.

**Figure 1:**
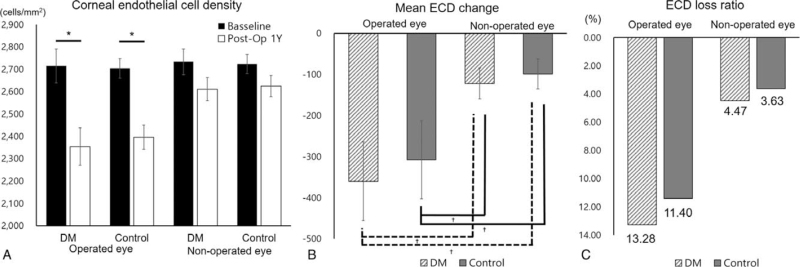
Changes in ECD after surgery. (A) Mean ECD preoperative and 1 year postoperatively. (B) Mean ECD change 1 year after surgery. (C) ECD loss percentage 1 year postoperatively. ^∗^*P* value by paired *t* test and ^†^*P* value by independent *t* test. DM = diabetic mellitus, ECD = endothelial cell density.

**Figure 2:**
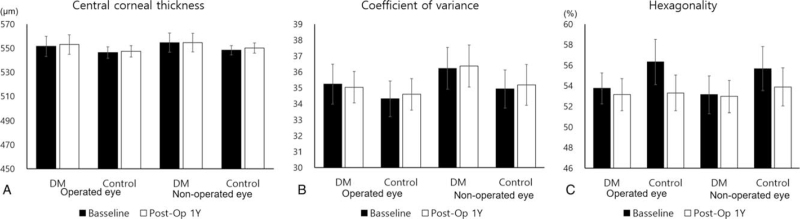
Changes in CV (A), hexagonality (B), and CCT (C) before and after surgery. The 3 parameters did not show any significant difference before and after surgery. CCT = central corneal thickness, CV = coefficient of variance, DM = diabetic mellitus.

The ECD in both the DM group and the control group decreased significantly compared to the preoperative ECD in the operated eye, but in the fellow non2operated eye, neither the DM nor the control group's ECD decreased significantly after 1 year (Fig. 1A). Mean ECD change was greatest in the DM group who underwent surgery, but there was no significant difference from the control group that underwent surgery (Fig. 1B). Mean ECD loss (%) was measured as 13.28%, 11.40%, 4.47%, and 3.63% in the operated DM, operated control, fellow DM, and fellow control groups, respectively (Fig. 1C). Meanwhile, hexagonality, CV, and CCT did not show any significant changes between the 2 groups before and after surgery (Fig. 2).

**Figure 3:**
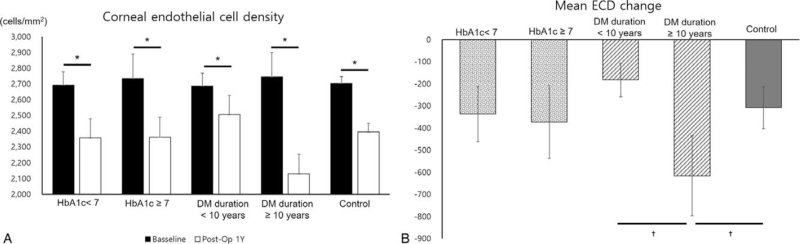
Subgroup ECD analysis based on disease duration and HbA1c concentration. (A) Mean ECD preoperative and 1 year postoperatively. (B) Mean ECD change 1 year after surgery. ^∗^*P* value by paired *t* test and ^†^*P* value by independent *t* test. DM = diabetic mellitus, ECD = endothelial cell density.

In the subgroup analysis performed on the operated eyes, 14 eyes in the low HbA1c group (<7%), 14 eyes in the high HbA1c group (≥7%), 15 eyes in the short DM duration (<10 years) group, and 13 eyes in the long DM duration group (≥10 years) were included. In all 4 subgroups, postoperative ECD was significantly reduced compared to preoperative ECD (Fig. 3A). Mean ECD change was significantly decreased in the long DM duration subgroup (≥10 years) compared to the short DM duration subgroup (<10 years) and the control group (Fig. 3B). However, there was no significant difference between the high HbA1c subgroup, the low HbA1c subgroup, and the control group.

In the correlation analysis, among age, HbA1c, glucose, DM duration, BUN, creatinine, anterior chamber depth, and axial length, only BUN correlated significantly with mean ECD change at 1 year postoperatively (*r* = −0.474, *P* = .011) (Table 2).

**Table 2:** Correlation between endothelial cell density change after phacoemulsification and preoperative systemic and ocular parameters in the diabetic group.

	ECD change	
	R value	*P* value
Age	−0.190	.333
Anterior chamber depth	0.313	.105
Axial length	0.213	.276
HbA1c	−0.198	.333
Glucose concentration	0.032	.871
Disease duration	−0.231	.236
BUN	−0.474	.011
Creatinine	−0.024	.904

BUN = blood urea nitrogen, ECD = endothelial cell density.

## 4 Discussion

In previous studies, various findings have been reported on the effect of diabetes on corneal endothelial cells, and in most studies, the ECD hexagonality decreased, and CV and CCT increased in diabetic patients when compared to nondiabetic individuals.^[21,27,28]^ This implies that diabetes is a potential risk factor for ECD loss after phacoemulsification. During cataract surgery, age, short axial length and shallow anterior chamber depth, high lens density, and large corneal incision size are factors known to affect corneal endothelial reduction.^[20,29,30]^ In this study, there was no statistically significant difference in preoperative age, lens density, axial length, and anterior chamber depth between the diabetes group and the control group. In addition, preoperatively, endothelial cell parameters were similar in both groups, indicating that no sampling bias was present (Table 1), and surgical factors were minimized by a similar surgical technique by 2 experienced surgeons using the same phacoemulsification machine in all cases.

Greater corneal endothelial cell loss was observed in diabetic subjects compared to nondiabetic subjects 1 year after phacoemulsification; however, there was no statistically significant difference. The ECD loss after cataract surgery reported in the DM group ranged from 5.95% to 29.07% in the literature (Table 3).^[6–17]^ Endothelial cell loss varies from study to study due to varying surgical techniques, different patient populations, and time points of corneal endothelial cell evaluation after surgery. Most previous studies reported that ECD loss in patients with diabetes is statistically significant after surgery compared to control groups. The duration of observation after cataract surgery varies from article to article, but in a report on ECD changes 3 months after surgery, Mathew et al^[8]^ reported a 19.24% ECD loss in diabetics compared to 16.58% in healthy controls. A study by Hugod et al^[2]^ showed a mean ECD loss of 6.2% in the diabetic group compared to 1.4% in the control group, and Yang et al^[9]^ reported the mean loss in ECD to be 18.36% in diabetic patients and 10.00% in nondiabetics at the 3-month follow-up. Langwinska-Wośko et al^[7]^ reported a significant discrepancy of 14% loss in diabetic vs 9% loss in nondiabetic patients in 2004. In our study, an average of 13.28% loss of ECD in the diabetic group and 11.40% in the control group after 1 year of phacoemulsification. These results are similar to those of previous studies. However, in our study, there was no statistically significant difference between the diabetes group and the control group. The reason for this is that, unlike previous studies, this study observed a relatively extended period of 1 year after surgery, and as a result, it is assumed that the central ECD recovered due to stabilization of the endothelium following a period of rearrangement.^[31,32]^ According to the recently reported studies of Ganesan et al^[13]^ and Fernández-Muñoz et al,^[14]^ ECD loss at 3 months after cataract surgery did not differ between diabetes and control groups. Further studies with large sample sizes are required.

**Table 3:** Summary of published results of ECD loss after phacoemulsification in diabetes and nondiabetes patients.

		Number of eyes			ECD loss (%)		
Authors	Year	Diabetes	Control	Follow-up	Diabetes	Control	Statistical differences
Morikubo et al^[6]^	2004	93	93	1 mo	7.20	3.20	Significant
Langwinska-Wośko et al^[7]^	2004	54	49	3 mo	15.38	11.33	Significant
Hugod et al^[2]^	2011	30	30	3 mo	6.20	1.40	Significant
Mathew et al^[8]^	2011	153	162	3 mo	19.24	16.58	Significant
Yang et al^[9]^	2011	126	112	3 mo	18.36	10.00	Significant
Misra et al^[10]^	2015	28	23	1 mo	0.90	0.09	Nonsignificant
He et al^[11]^	2017	66	67	1 mo	15.00	6.60	Nonsignificant
Sahu et al^[12]^	2017	60	60	3 mo	5.95	4.52	Significant
Ganesan et al^[13]^	2019	80	80	3 mo	16.60	18.19	Nonsignificant
Fernández-Muñoz et al^[14]^	2019	21	21	3 mo	29.07	13.71	Nonsignificant
Maadane et al^[15]^	2019	32	32	3 mo	6.41	4.59	Significant
Khalid et al^[16]^	2019	80	80	2 mo	14.88	9.86	Significant
Kudva et al^[17]^	2020	54	52	3 mo	27.50	18.3	Significant
Our study	2021	28	37	12 mo	13.28	11.40	Nonsignificant

ECD = endothelial cell density.

In this study, unlike other previous studies, the fellow eye not receiving surgery was also evaluated in order to confirm the natural decrease in ECD. The ECD loss for 1 year in the fellow eye was 4.47% in DM patients and 3.63% in the control group, and there was no significant difference in corneal endothelial cell parameters between the 2 groups before and after surgery. In general, ECD has its highest density at birth at 7500 cells/mm^2^, and it is known to gradually decrease by roughly 0.5% per year in the second decade of life.^[33]^ The results of this study showed a greater ECD loss per year (%) than the known values. This is likely to be higher than the typical rate of loss during lifetime because the mean age of the patients included in this study was over 60 years old.

In the subgroup analysis, the ECD loss in the long DM duration subgroup was significantly higher than that in the short DM duration subgroup and the control group. Few studies have reported ECD loss after cataract surgery according to disease duration and glucose control. Unlike the results of this study, Kudva et al^[17]^ reported that there was no difference between groups when DM patients were divided based on a disease duration of 10 years, and there was no association with HbA1c levels. They explained that the reason for these results was that type 2 diabetes had no impact on corneal cell density in subjects with good glycemic status.^[17]^ However, another study reported that DM of more than 10 years was associated with more significant natural ECD changes.^[19]^ This is because the longer the disease duration, the higher the probability of chronic microvascular complications due to chronic exposure to hyperglycemic conditions, and the corneal endothelium becomes vulnerable.^[34]^ Despite the absence of significant differences between the diabetic and control groups with respect to preoperative ECD, postoperative ECD and ECD loss, diabetic patients in the current study demonstrated more ECD loss postoperatively compared to nondiabetic patients. Therefore, diabetes duration may be considered to affect postoperative ECD loss and further studies are needed.

The large ECD loss in diabetic patients is because their endothelial cells are vulnerable to trauma caused by cataract surgery. Potential theories for increased vulnerability include oxidative stress due to chronic overall ischemia, accumulation of advanced glycation end products occurring in diabetes, and underlying corneal neuropathy, making the eye more susceptible to the damaging impact of surgery.^[4,5,10,35]^ In addition, diabetes also impairs the activity of the Na^+^K^+^-ATPase enzyme in endothelial cells. These mechanisms can reduce the cornea's ability to control corneal hydration.^[35]^ There is also a report that corneal endothelial cells are easily damaged in case of the lower initial endothelial cell count.^[19–21]^

Meanwhile, CV, hexagonality, and CCT did not show statistically significant differences between the DM and control groups both before and after surgery. CV is an indicator of the uniformity of the size of endothelial cells, which indicates the activity of the repair and healing mechanism of the endothelium after damage. Previous studies demonstrated nonsignificant differences in CV changes in diabetic patients after cataract surgery.^[2,6,19]^ However, even though it was not statistically significant, the preoperative and postoperative CV values were larger in the DM group than in the control group. Surgeons should be aware that the recovery of endothelial cells may be slower and less significant in diabetics. Hexagonality indicates the variability in hexagonal cell shape, such as CV, it means the healing response after damage. In this study, hexagonality also decreased at 1 year postoperatively in the DM and control groups, but the difference was not statistically significant. Various results have been reported previously. Lee et al^[19]^ and Morikubo et al^[6]^ reported that there was no difference in hexagonality between the DM and control groups, and Hugod et al^[2]^ showed a significant decline in the percentage of hexagonal cells only among diabetic patients. One year is a sufficient amount of time for the corneal endothelial cells to recover, and therefore, it is believed that there was no significant difference in this study. CCT was used as a marker for endothelial function. Similar results were found in previous studies. Hugod et al^[2]^ and Elbassiouny et al^[26]^ reported that there were no differences in CCT between diabetic and nondiabetic patients after surgery. This is because there is ECD loss after cataract surgery, but this is not sufficient to affect corneal endothelial cell function.

In this study, we determined a significant negative correlation between postoperative ECD loss and BUN in patients in the diabetic group. BUN is an indicator of the gross index of glomerular function. In previous studies on its effect on corneal endothelial cells, BUN was significantly higher in DM patients than in the control group, but it did not affect the corneal endothelium, and other study reported that the urinary albumin-creatinine ratio was correlated with ECD.^[36,37]^ Regarding renal function and corneal endothelial cell status, Ohguro et al^[38]^ reported that polymegethism and pleomorphism were increased in patients with chronic renal failure. The amount of urea in the aqueous humor is known to increase in proportion to blood urea, and oxidized glutathione is increased in the aqueous humor in patients with renal failure, which is described as urea, and may be toxic to the corneal endothelium.^[39,40]^ However, since our sample size was small, additional research and analyses are needed.

This study is limited by its small sample size and its retrospective nature; therefore, our findings cannot be generalized to all patients who undergo phacoemulsification in diabetes. In addition, during phacoemulsification, trauma by ultrasound energy plays a significant role in ECD loss. In this study, ultrasound energy could not be analyzed. Despite these limitations, unlike previous studies, our study reported ECD change over a relatively long term of 1 year in diabetes after phacoemulsification, and through subgroup analysis, it was confirmed that ECD loss was large when the disease duration was 10 years or longer. Based on this, our results add further evidence to the literature regarding the effect of DM on ECD loss after phacoemulsification.

## 5 Conclusion

In conclusion, there was a statistically significant decrease in ECD 1 year after surgery in both the diabetic and control groups. In the subgroup analysis, the long-duration DM group (≥10 years) showed a significantly greater postoperative ECD loss than the control group and short-duration DM group (<10 years) and BUN showed correlation with postoperative ECD decreases. Therefore, in DM patients with a duration of 10 years or more or with high serum BUN, the possibility of ECD reduction after cataract surgery should be considered when planning cataract surgery.

## Author contributions

**Conceptualization:** Tae Gi Kim.

**Data curation:** Jin-Ho Joo.

**Formal analysis:** Jin-Ho Joo, Tae Gi Kim.

**Funding acquisition:** Tae Gi Kim.

**Investigation:** Tae Gi Kim.

**Methodology:** Jin-Ho Joo, Tae Gi Kim.

**Writing – original draft:** Jin-Ho Joo, Tae Gi Kim.

**Writing – review & editing:** Tae Gi Kim.
